# Pre-transplant CD45RC expression on blood T cells differentiates patients with cancer and rejection after kidney transplantation

**DOI:** 10.1371/journal.pone.0214321

**Published:** 2019-03-29

**Authors:** Anne-Sophie Garnier, Martin Planchais, Jérémie Riou, Clément Jacquemin, Laurence Ordonez, Jean-Paul Saint-André, Anne Croue, Abdelhadi Saoudi, Yves Delneste, Anne Devys, Isabelle Boutin, Jean-François Subra, Agnès Duveau, Jean-François Augusto

**Affiliations:** 1 LUNAM Université, Angers, France; 2 CHU Angers, Service de Néphrologie-Dialyse-Transplantation, Angers, France; 3 MINT, UNIV Angers, INSERM 1066, CNRS 6021, Université Bretagne Loire, IBS- CHU, Angers, France; 4 INSERM U1035, BMGIC, Immuno-dermatology ATIP-AVENIR, University of Bordeaux, Bordeaux, France; 5 Université de Toulouse, Centre de physiopathologie de Toulouse Purpan, Toulouse, France; 6 CHU Angers, Laboratoire d’anatomopathologie, Angers, France; 7 CRCINA, INSERM, Université de Nantes, Université d’Angers, Angers, France; 8 LabEx IGO “Immunotherapy, Graft, Oncology”, Angers, France; 9 Laboratoire HLA, Etablissement Français du Sang Pays de Loire, Angers, France; 10 Centre de Sante, Etablissement Français du Sang Pays de Loire, Angers, France; University of Toledo, UNITED STATES

## Abstract

**Background:**

Biological biomarkers to stratify cancer risk before kidney transplantation are lacking. Several data support that tumor development and growth is associated with a tolerant immune profile. T cells expressing low levels of CD45RC preferentially secrete regulatory cytokines and contain regulatory T cell subset. In contrast, T cells expressing high levels of CD45RC have been shown to secrete proinflammatory cytokines, to drive alloreactivity and to predict acute rejection (AR) in kidney transplant patients. In the present work, we evaluated whether pre-transplant CD45RC^low^ T cell subset was predictive of post-transplant cancer occurrence.

**Methods:**

We performed an observational cohort study of 89 consecutive first time kidney transplant patients whose CD45RC T cell expression was determined by flow cytometry before transplantation. Post-transplant events including cancer, AR, and death were assessed retrospectively.

**Results:**

After a mean follow-up of 11.1±4.1 years, cancer occurred in 25 patients (28.1%) and was associated with a decreased pre-transplant proportion of CD4^+^CD45RC^high^ T cells, with a frequency below 51.9% conferring a 3.7-fold increased risk of post-transplant malignancy (HR 3.71 [1.24–11.1], p = 0.019). The sensibility, specificity, negative predictive and positive predictive values of CD4^+^CD45RC^high^<51.9% were 84.0, 54.7, 89.8 and 42.0% respectively. Confirming our previous results, frequency of CD8^+^CD45RC^high^ T cells above 52.1% was associated with AR, conferring a 20-fold increased risk (HR 21.7 [2.67–176.2], p = 0.0004). The sensibility, specificity, negative predictive and positive predictive values of CD8^+^CD45RC^high^>52.1% were 94.5, 68.0, 34.7 and 98.6% respectively. Frequency of CD4^+^CD45RC^high^ T cells was positively correlated with those of CD8^+^CD45RC^high^ (p<0.0001), suggesting that recipients with high AR risk display a low cancer risk.

**Conclusion:**

High frequency of CD45RC^high^ T cells was associated with AR, while low frequency was associated with cancer. Thus, CD45RC expression on T cells appears as a double-edged sword biomarker of promising interest to assess both cancer and AR risk before kidney transplantation.

## Introduction

Despite significant therapeutic advancements in immunosuppressive drug regimens, acute rejection (AR) remains a severe complication of kidney transplantation which is associated with the development of chronic allograft nephropathy and premature graft loss [1]. Alloreactive T cells, including CD4^+^ and CD8^+^ T cells, have a critical role in AR [2]. Actually, induction (ie, anti-thymocyte globulins, anti-IL2R mAb) and maintenance regimens (ie anticalcineurin, antiproliferative agents) target T cells without specificity for T cell subsets [3]. Thus, identifying among CD4^+^ and CD8^+^ T cells, the specific subsets that drive alloreactivity constitutes an objective for the development of targeted therapies able to induce and maintain long-term allograft tolerance. Among T cell subsets, regulatory T (Treg) cells play a central role in the maintenance of tolerance to auto/allo-antigens by suppressing auto/allo-reactive T cells [4, 5]. In support, Treg cell proportion or their absolute number, as well as their functional properties, have been found altered in graft recipients that developed AR when compared to those of tolerant patients [6–8].

The identification of patients with high risk, or conversely with low risk of AR, is of critical importance to tailor immunosuppressive treatment intensity. Indeed, long-term exposition to immunosuppressive drugs is not only associated with cancer risk, but also with cardiovascular disease and infection risks. These complications represent the main causes of death in transplanted patients [9, 10].

Focusing on cancer, as compared to the general population, its relative risk in kidney transplant patient is increased by 2 to 4-fourfold for solid cancers [11]. However, the relative risk is variable between cancer types with non-melanoma skin cancer and posttransplant lymphoproliferative disorders being increased by by 10 to 40 times and 4 to 16 times, respectively [11, 12]. Its development in kidney transplant recipients has been related to the intensity of immunosuppressive load, but also to pre-transplant factors, such as older age, past history of malignancy and exposition to several other susceptibility factors (ie, viruses, UV)[13]. However, taken individually, these risk factors are poorly predictive of cancer development at the individual level. Interestingly, to elucidate immune factors associated with cancer risk in kidney transplant patients, Hoppe et al observed an increased count and proportion of circulating Treg cells in kidney transplant recipients that developed cancer [14]. Whether modifications of Treg cell compartment was a consequence or a causal factor of cancer remains to be elucidated [15]. Nevertheless, these observations open the question of whether specific immune profiles favor cancer development. Supporting this view, Th2 type cells infiltrating tumors and Th2 cytokines have been reported to favor both tumor occurrence and tumor growth [16].

Finally, in kidney transplant patients, AR risk has been associated with Th1/Th17 cytokine secreting cells, while cancer has rather been related to an increase in regulatory T cell compartment and Th2 cytokine milieu [17, 18]. On the other hand, Treg cells are associated with a tolerant profile and constitutes the background of several clinical studies aimed to reduce AR occurrence by using Treg cell compartment manipulation [19]. Finally, this immune dilemma allows to hypothesize that immune profiles before transplantation may be associated with post-transplant AR or cancer and may be used as a biomarker. In this view, which we acknowledge may appear in some extend simplistic, patients at low AR risk would benefit of an alleviated immunosuppression which would also reduce the cancer risk, and conversely.

CD45 is a transmembrane protein tyrosine phosphatase heavily expressed on hematopoietic cells, especially on T and B cells, critical for signal transduction by regulating kinases of the Src-family (Lck in T cells or Lyn, Fyn and Lck in B cells) [20, 21]. Four CD45 isoforms (RO, RA, RB, RC) resulting from an alternative splicing of 3 exons and differing by size and charge of their extracellular domains are expressed in humans [20]. Even though CD45 alternative splicing is highly regulated, the functions of the different isoforms remain unclear. CD45RA and CD45RB isoforms are mainly expressed on naive T cells, whereas CD45RO is preferentially expressed on memory T cells [21, 22].

The CD45RC isoform is highly expressed on human naive T and B cells, as well as on NK cells and activated CD16^+^ monocytes [22]. Focusing on human T cells, a bimodal pattern of expression is observed on CD4^+^ T cells (high and low expression), while a trimodal one is observed on CD8^+^ T cells (low, intermediate and high) [23, 24]. These expression patterns determine CD45RC T cell subsets with different cytokine secretion profiles after in vitro polyclonal stimulation. Indeed, CD45RC^high^ T cell subset mainly secrete Th1 cytokines, while CD45RC^low^ subset mainly secrete regulatory cytokines [23, 24]. Importantly, the expression of CD45RC on T cells is highly variable between individuals, is genetically determined and has been shown independent of age in healthy subjects [23–25].

In a previous work, we demonstrated that the level of CD45RC expression at the surface of blood CD8^+^ T cells of candidate patients to kidney transplantation was associated with the risk of developing AR after transplantation [23]. Indeed, by studying a cohort of 89 kidney transplant recipients with a median follow-up of 5 years, we observed that a pre-transplant proportion of CD8^+^CD45RC^high^ T cells above 54.7% conferred randomly a 6-fold increased risk of developing AR after adjustment on age. The long-term follow-up of this cohort of patients who were transplanted between 1999 and 2004, at the University Hospital of Angers, France, allowed us to analyze the relationship between CD45RC subsets and cancer occurrence. Thus, in the present work, we hypothesized that patients that developed cancer had a lower CD45RC^high^ T cell proportion as compared to patients that did not develop cancer during the follow-up.

We show here that a low pre-transplant proportion of CD45RC^high^ T cells is associated with a higher risk of developing cancer after kidney transplantation. We also confirm, in this long-term analysis, that patients with a high pre-transplant proportion of CD45RC^high^ expression on T cells have an increased risk of AR. Finally, we were able to differentiate two groups of patients, one with a high risk of cancer and the other with a high risk of AR based on CD45RC expression on T cells. Thus, these data also support that specific pre-transplant immune profiles constitute a risk factor for post-transplant complications.

## Material and methods

### Study design and end-points

The present study constitutes a long-term analysis of the original cohort study from Ordonnez et al whose results were published in 2013 [23]. The original population was constituted of 89 consecutives first time kidney transplanted recipients in Angers University Hospital, between 1999 and 2004. All patients gave their written consent to participate. In this study, patients with panel reactive antibodies > 20% were excluded from inclusion. The study was approved by the Medical Ethics Committee of the University Hospital Center of Angers (2009/10).

The primary objective of the present study was to analyze the relationship between CD45RC expression on T cell subsets and cancer within the same cohort of transplant patients after a long-term follow-up. The secondary objective was to analyze whether the relationship between CD45RC expression on T cells and AR risk remained significant in the long-term follow-up.

### Immunosuppressive regimens

The details of the immunosuppressive treatments have been detailed elsewhere [23]. Briefly, the induction regimen included one methylprednisolone bolus of 500 mg alone or in association with either two injections of basiliximab (Simulect; Novartis Pharma, Switzerland) (20 mg on day 0 and day 4 posttransplantation), or antithymocyte antibodies administration (Thymoglobuline; Genzyme, France) during the first 3 to 7 days posttransplantation. All patients received prednisone (1 mg/kg/day) with a progressive tapering and discontinuation at the end of month 5 post-transplant unless occurrence of more than one acute rejection episode. Maintenance immunosuppressive regimen relied on mycophenolate mofetil (Cellcept, Roche, France) and tacrolimus (Prograf, Fujisawa, Japan). In patients that did not experience AR, mycophenolate mofetil was withdrawn at month 4 posttransplant, and tacrolimus monotherapy was used as maintenance regimen after month 6 posttransplant.

### Data collection and definitions

Characteristics of the study population have been previously published [23]. The long-term data, including clinical events and biological data, were collected retrospectively by a systematic screening of patients’ medical records until last follow-up, graft loss or patient death. Diagnosis of AR episodes was based on conventional clinical and laboratory criteria and confirmed by histological examination of graft biopsy. For the study purpose, all graft biopsies were reviewed by an experimented kidney pathologist and scored according Banff 2013 classification [26]. AR episodes which were clinically suspected and treated were also considered in the study. Cancers were collected and classified as non-melanoma skin cancers, solid cancers and post-transplant lymphoproliferative disease (PTLD). Cases of graft loss and patient death, as well as causes of death were registered.

### Donor specific anti-HLA antibody determination

Donor specific anti-HLA antibodies (DSA) against HLA-A, -B, -Cw, -DR, -DQ, -DP were tested retrospectively using historical serum sampled at the time of graft biopsy. DSA were detected using single antigen flow assays (One Lambda Inc., Canoga Park, CA) on a Luminex plateform (Austin, Tx). A normalized MFI > 1000 was considered significant.

### Flow cytometry

Expression of CD45RC on T cells of patients of the cohort has been reported previously by Ordonnez et al [23]. Briefly, CD45RC expression was determined using flow cytometry in pretransplant peripheral blood mononuclear cells, which were frozen in liquid nitrogen before renal transplantation. Data were collected either on a FACS-Calibur (BD Biosciences) cytometer using the CELLQuest software (BD Biosciences) for analysis, or on a LSR-II (BD Biosciences) cytometer using the DIVA software (BD Biosciences) for analysis. S1 Fig illustrates the gating strategy for cell population analyses.

### Statistical analysis

Data were expressed as mean ± SD for quantitative variables and number (percentage) for qualitative variables. Categorical and continuous data were analyzed with χ^2^ or Fischer’s exact test and Mann-Whitney U (or Kruskal-Wallis) tests, respectively. The predictive values of CD45RC subset frequency were analyzed and compared using receiver operating characteristics (ROC) curves. Subsequently, cut-off values were determined by using the Youden index. Kaplan-Meyer method was used to analyze event-free survivals according to predetermined cut-off values of CD45RC subset frequencies. A log-rank test was used to compare survival curves. Correlations were analyzed using Spearman’s rank correlation test. Multivariate cox models were used to analyze the association between CD45RC subset frequencies and events. Results are reported as hazard ratio (HR) with 95% CIs. All *p* values were two-sided and *p* value lower than 0.05 was considered statistically significant. Statistical analysis was performed using Graphpad Prism and SPSS software 22.0.

## Results

### Baseline population characteristics and post-transplant events

The main characteristics of the population were previously reported [23]. In summary, the cohort included 89 consecutives and first time transplanted patients, predominantly males (75.5%) with a mean age of 48.1±15.2 years at transplantation. Grafts came from deceased donors and the mean cold ischemia time was 18.3±5.9 hours. Most patients received induction therapy (91%), with anti-thymocyte globulins (62%) or basiliximab (29%). Tacrolimus-based regimens were used in 85% of patients and 64% were treated with tacrolimus monotherapy after month 6 post-transplant. These data are summarized in S1 Table.

The mean follow-up of the cohort was 11.1±4.1 years (Table 1) and none patient was lost from follow-up. During the analysis period, cancer was diagnosed in 25 patients (28.1%), predominantly non-melanoma skin cancers. Thirteen patients (14.6%) developed at least 1 non-melanoma skin cancer, six (6.7%) developed both skin and solid cancers, 4 (4.5%) solid cancers and 2 (2.2%) post-transplantation lymphoproliferative disorders (PTLD). The mean delay to first cancer was 6.7±4.3 years. Acute rejection episodes were updated, 18 patients (20.1%) experienced AR (23 episodes) that occurred after a mean delay of 3.4±3.8 years from transplantation. Rejection episodes were T cell-mediated (TCMR) in 7 cases and antibody-mediated (AMR) in the 7 other cases. All but one AMR episodes were associated with DSA.

**Table 1:** Post-transplant events.

Mean follow-up, years	11.1 ± 4.1
Acute rejection	
Number of patients, n (%)	18 (20.1)
Number of episode, n	23
Mean delay to first AR (years)	3.4 ± 3.8 [0–14.1]
Histologically proved AR, n	14
TCMR	7
AMR	7
Non histologically proved AR, n	4
Cancer, number of patients (%)	
All types	25 (28.1)
Skin	17 (19.1)
Solid	10 (11.2)
PTLD	2 (2.2)
Mean delay to cancer (years)	
All types	6.7 ± 4.3 [0.4–15.7]
Skin	7.1 ± 3.8 [2.3–15.1]
Solid/PTLD	5.0 ± 4.4 [0.4–15.7]
Death, n (%)	14 (15.7)
Mean delay (years)	7.0 ± 4.0 [0.1–14.3]
Graft loss, n (%)	20 (22.5)
Mean delay (years)	5.9 ± 4.3 [0.1–12.8]
Year 1 post-transplant biological results	
Serum creatinine, (μmol/L)	129.4 ± 34.7
GFR, (mL/min/1.73m2)*	55.9 ± 16 [23.6–110]
Proteinuria, (g/day)	0.21 ± 0.27
Last follow-up biological results	
Serum creatinine, (μmol/L)	147.0 ± 83.2
GFR, (mL/min/1.73m2)*	54.4 ± 24 [11.2–116]
Proteinuria, (g/day)	1.05 ± 2.9

* In patients followed at the indicated time

AR, acute rejection; GFR, glomerular filtration rate; PTLD, posttransplant lymphoproliferative disorder; TCMR, T cell mediated rejection; AMR, Antibody mediated rejection.

Four AR episodes were clinically diagnosed and treated without histological confirmation. Twenty (22.5%) patients lose their graft after a mean delay of 5.9±4.3 years and the 15-years estimated graft survival was 70.2% (S2A Fig). Death occurred in 14 (15.7%) patients at a mean delay of 7±4.0 years. Kaplan Meyer analysis showed an estimated patient survival of 79.2% at 15 years of follow-up (S2B Fig).

### Association between CD45RC expression on T cells and post-transplant events

In a first set of analysis, we compared the mean frequency of pre-transplant CD45RC patterns of expression in CD4^+^ T cells (CD45RC high or low) and in CD8^+^ T cells (CD45RC low, int or high) according to post-transplant events (Table 2). In line with our previous results, we observed that the pre-transplant frequency of CD8^+^CD45RC^high^ T cells was significantly higher in patients that experienced AR as compared to patients that did not present AR [23]. Accordingly, the proportions of CD8^+^CD45RC^low^ and of CD8^+^CD45RC^int^ T cells were significantly lower in patients with AR. The frequency of CD4^+^CD45RC^high^ T cells was also higher in AR patients as compared to patients without AR.

**Table 2:** Frequency of CD4^+^ and CD8^+^CD45RC subsets according presence or absence of post-transplant complications. Results are expressed as the % of subset among CD4^+^ or CD8^+^ T cells.

	Yes	No	*p*
**Acute rejection, n**	18	71	
CD4 CD45RC ^high^	55.3 ± 10.8	45.2 ± 15.7	**0.012**
CD8 CD45RC ^high^	63.2 ± 9.8	45.0 ± 16.2	**<0.001**
CD8 CD45RC ^int^	23.9 ± 8.1	33.6 ± 11.3	**0.001**
CD8 CD45RC ^low^	12.9 ± 3.9	21.8 ± 11.3	**0.001**
**Cancer, all types, n**	25	64	
CD4 CD45RC ^high^	39.1 ± 13.9	50.4 ± 14.8	**0.001**
CD8 CD45RC ^high^	41.7 ± 16.2	51.4 ± 13.3	**0.014**
CD8 CD45RC ^int^	35.8 ± 13.8	30.0 ± 10.0	**0.028**
CD8 CD45RC ^low^	22.4 ± 10.2	19.1 ± 11.1	0.189
**Death, n**	14	75	
CD4 CD45RC ^high^	37.2 ± 13.9	50.0 ± 14.6	**0.001**
CD8 CD45RC ^high^	39.9 ± 15.4	51.1 ± 16.5	**0.008**
CD8 CD45RC ^int^	35.8 ± 11.4	30.4 ± 11.2	0.064
CD8 CD45RC ^low^	24.3 ± 12.5	18.9 ± 10.1	0.051

We next applied the same kind of analysis to cancers. Frequency of CD4^+^CD45RC^high^, as well as those of CD8^+^CD45RC^high^, were significantly lower in patients with cancer as compared to patients without cancer (Table 2). We also compared cell frequencies according to cancer subtype (skin cancers and solid cancers/PTLD). The difference remained significant when analysis was done according to cancer subtype for CD4^+^CD45RC^high^ T cells frequency, while CD8^+^CD45RC^high^ T cell frequency was no longer different between patients with or without solid cancer/PLTD (S2 Table). Frequencies of CD45RC^high^ subsets of CD4^+^ and CD8^+^ T cells were also significantly lower in patients that died during the follow-up (Table 2). S3 Fig shows the frequency of CD4^+^ and CD8^+^CD45RC in patients that did not developed cancer or AR (n = 48), and those that developed cancer (n = 16), AR (n = 23) and both (n = 2).

### Factors associated with cancer and predictive value of CD45RC expression on T cells for cancer prediction

We first studied factors associated with cancer using univariate analysis (Table 3). We observed that patients with cancer were significantly older at transplantation than patients that did not developed cancer (p = 0.001). No difference was observed between groups according to gender, cold ischemia time, number of HLA mismatches, donor age, and immunosuppressive regimens.

**Table 3:** Univariate analysis of factors associated with cancer occurrence.

	Cancer (n = 25)	No cancer (n = 64)	*p*
**Baseline characteristics**			
Sex (M/F)	21/4	48/16	0.414
Age (years)	56.5 ± 10.9	44.9 ± 15.5	**0.001**
**History of transplantation**			
Pre-transplant dialysis, n (%)	22 (88.0)	47 (73.4)	0.168
Donor age, years	45.6 ± 15.8	39.2 ± 17.5	0.115
Cold ischemia time (hours)	19.9 ± 4.8	17.6 ± 6.2	0.101
HLA mismatch (ABDR)	3.7 ± 1.1	3.9 ± 1.3	0.595
**Immunosuppressive regimens**			
Induction therapy (none/basiliximab/ATG)	0/11/14	2/21/41	0.450
Tac monotherapy, n (%)	17 (68.0)	40 (62.5)	0.806
Tacrolimus-based regimen, n (%)	19 (76.0)	57 (89.1)	0.179

**Fig 1:**
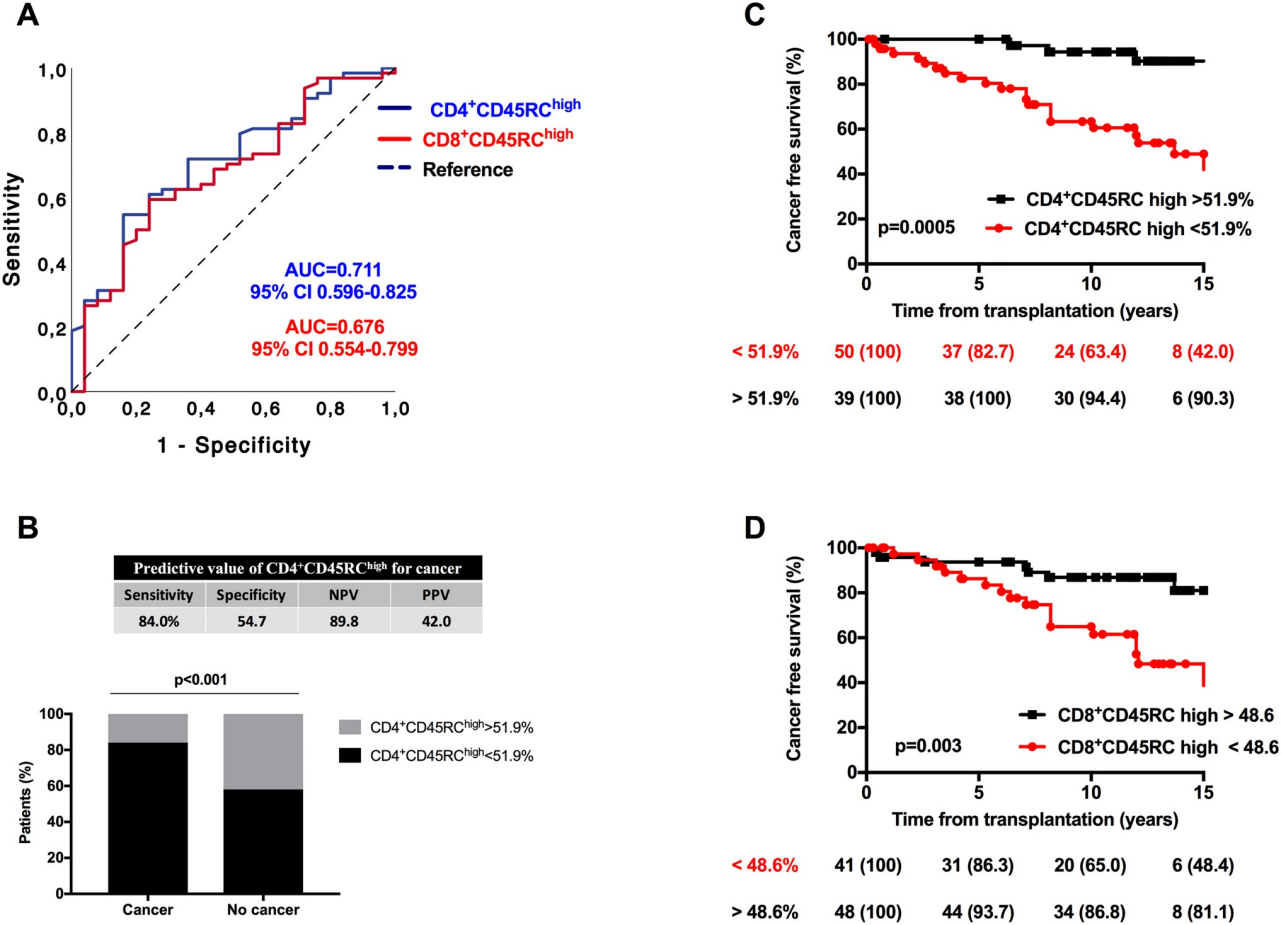
Predicting value of CD4^+^ and CD8^+^CD45RC subsets for cancer and death. (**A**), ROC curve analysis of CD4^+^CD45RC^high^ (blue line) and CD8^+^CD45RC^high^ (red line) T cells for cancer prediction. (**B**), Frequency of patients with a positive test for CD4^+^CD45RC^high^ as determined by ROC analysis (threshold 51.9%) according to presence or absence of cancer. (**C&D**), Survival free of cancer according to proportion of (**C**) CD4^+^CD45RC^high^ and (**D**) CD8+CD45RC^high^ T cells. Threshold values were determined according to ROC curve analysis.

We next used ROC curve to study the predicting value of CD4^+^ and CD8^+^CD45RC cell frequencies for cancer and to determine the best cut-off values for cancer prediction (Fig 1). The analysis revealed that the proportion of both CD4^+^ and CD8^+^CD45RC^high^ T cells were predictive of cancer, with an AUC of 0.711 ([0.596–0.774], p = 0.002) and of 0.676 ([0.554–0.799], p = 0.01), respectively (Fig 1A). A cut-off of T cell frequency below 51.9% for CD4^+^CD45RC^high^ (sensibility 84.0%, specificity 54.7%, positive predictive value 42.0%, negative predictive value 89.8%, Fig 1B) and 48.6% for CD8^+^CD45RC^high^ appeared as the best thresholds for cancer prediction (sensibility 68.0%, specificity 50.0%, positive predictive value 41.0%, negative predictive value 75.0%). Kaplan Meier analysis showed that patients with low pretransplant proportions of CD4^+^CD45RC^high^ (Fig 1C) or CD8^+^CD45RC^high^ T cells (Fig 1D) had a significantly lower survival without cancer as compared to patients with high proportions. Multivariate cox analysis showed that frequency of CD4^+^CD45RC^high^ below 51.9% was significantly associated with cancer after adjustment on age, gender, and induction therapy (HR 3.71, p = 0.019, Table 4).

**Table 4:** Multivariate cox analysis for prediction of cancer.

	Multivariate Cox models	HR	95% CI	*P*
**CD4**^**+**^**CD45RC**^**high**^	CD4 CD45RC^high^ (<51.9%)	3.71	1.24–11.1	**0.019**
	Age at transplantation*	1.05	1.02–1.15	**0.005**
	Gender (male)	1.57	0.51–4.85	0.429
	Induction (ATG)	1.67	0.66–4.24	0.282
**CD8**^**+**^**CD45RC**^**high**^	CD8 CD45RC^high^ <48.6%	1.99	0.80–4.96	0.136
	Age at transplantation*	1.05	1.02–1.09	**0.003**
	Gender (male)	1.42	0.65–4.28	0.284
	Induction (ATG)	1.67	0.80–4.96	0.136

* per year increment

### Predictive value of CD45RC expression on T cells for AR and death prediction

Using an univariate analysis, we observed that patients with AR were significantly younger than patients that did not developed AR (p<0.001). No difference was observed between the groups according to gender, cold ischemia delay, number of HLA mismatches and immunosuppressive regimens. Donor age tended to be lower in patients that experienced AR (S3 Table).

**Fig 2:**
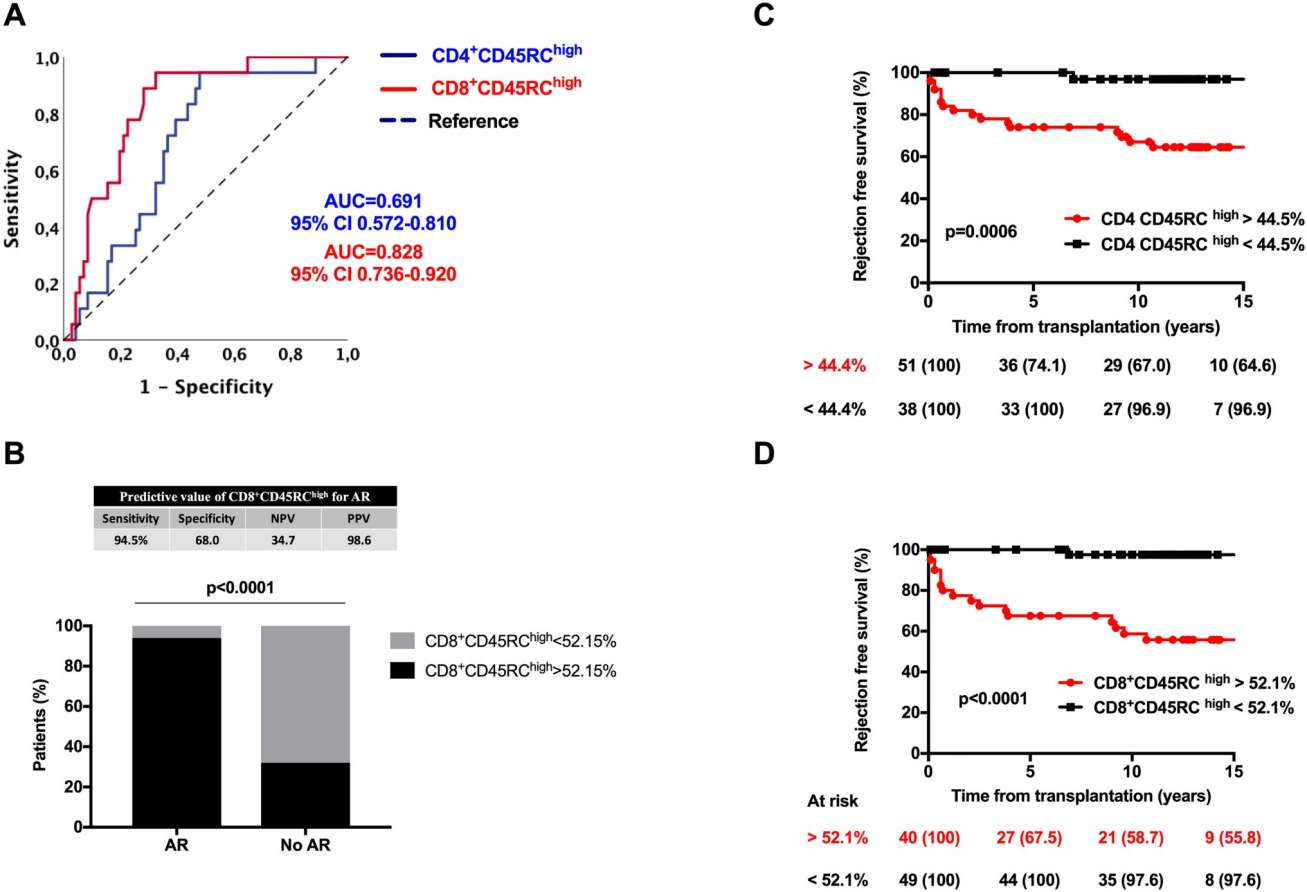
Predicting value of CD4^+^ and CD8^+^CD45RC subsets for acute rejection (AR). (**A**), ROC curve analysis of CD4^+^CD45RC^high^ (blue line) and CD8^+^CD45RC^high^ (red line) T cells. (**B**), Frequency of patients with a positive test for CD8^+^CD45RC^high^ as determined by ROC analysis (threshold 52.15%) according to presence or absence of AR. (**C&D**), Survival free of acute rejection according to proportion of (**C**) CD4^+^CD45RC^high^ and (**D**) CD8+CD45RC^high^ T cells. Threshold values were determined according to ROC curve analysis.

Applying a similar analysis than for cancer prediction (Fig 2), we observed that the proportion of CD8^+^CD45RC^high^ T cells was highly predictive of AR (p<0.001). Although less significantly, the proportion of CD4^+^CD45RC^high^ T cells was also associated with AR (p = 0.013). Among CD8^+^ T cells, the CD45RC^high^ proportion had the best predictive value with an AUC of 0.828 (p<0.001). Based on ROC curve analysis, a threshold above 52.1% for CD8^+^CD45RC^high^ expression appeared as the best cut-off value (sensibility 94.5%, specificity 68.0%, positive predictive value 34.7%, negative predictive value 98.6%, Fig 2A & 2B).

Based on predetermined cut-off values, we next analyzed survival free of AR according to frequency of CD4^+^CD45RC^high^ and CD8^+^CD45RC^high^ frequencies (Fig 2C & 2D). Patients with high proportion of CD4^+^CD45RC^high^ (Fig 2C) and CD8^+^CD45RC^high^ (Fig 2D) had a lower survival free of AR as compared to patients with lower frequencies of CD45RC^high^ subsets (p = 0.0006 and p<0.0001, respectively). Using a multivariate cox model, we observed that frequency of CD8^+^CD45RC^high^ above 52.1% was significantly associated with AR after successive adjustment on age, gender, and induction therapy (HR 21.7, p = 0.004). CD4^+^CD45RC^high^ frequency above 44.5% was also significantly associated with AR, but at a lower extend, after adjustment on age, gender and induction therapy (HR 9.88, p = 0.032). These data are reported in S4 Table.

Finally, we analyzed the relation between CD45RC expression and patient death. ROC curve analysis showed a significant predictive value of CD4^+^CD45RC^high^ and CD8^+^CD45RC^high^ T cell frequency (AUC = 0.771, p = 0.001 and AUC = 0.710, p = 0.013, S4 Fig). A cut-off of T cell frequency below 38.8% for CD4^+^CD45RC^high^ and 49.1% for CD8^+^CD45RC^high^ appeared as the best thresholds for death prediction. However, in the multivariate analysis, frequencies of CD45RC subsets according to these cut-offs were no longer associated with death after adjustment on other variables (S5 Table).

### Association between expression of CD45RC on CD4^+^ and CD8^+^ T cells

**Fig 3:**
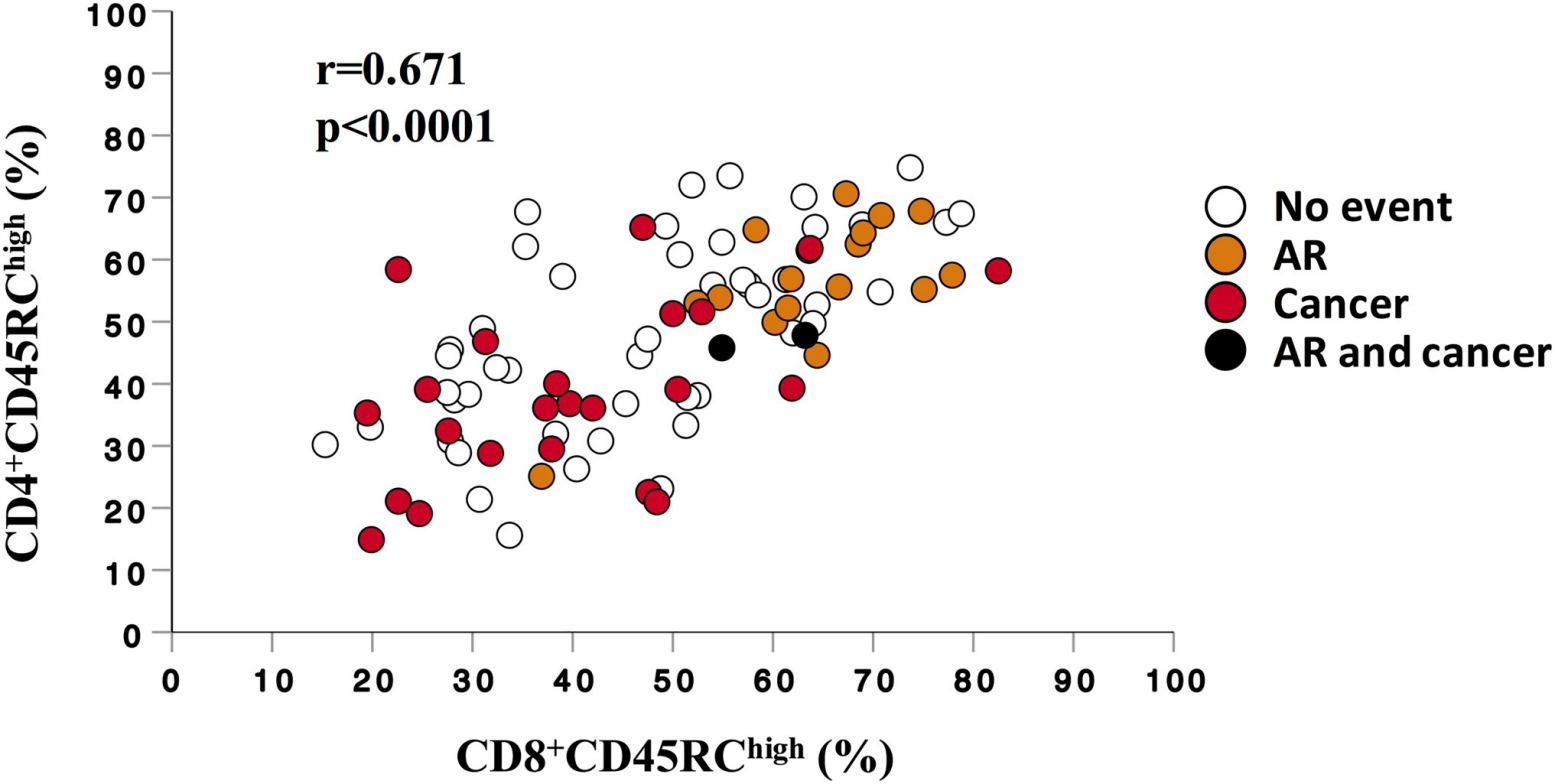
Correlation between frequencies of CD4^+^CD45RC^high^ and CD8^+^CD45RC^high^. Each dot represents a patient and reports observed percentage of CD45RC^high^ on CD4^+^ (y-axis) and CD8^+^ T cells (x-axis). White dots represent patients that did not developed cancer and AR, orange dots represent patients that developed AR, red dots represent patients that developed cancer and black dots represent patients that developed both AR and cancer. Statistical analysis was done using Spearman’s rank correlation test.

Given that a high frequency of CD8^+^CD45RC^high^ was associated with AR occurrence and that a low frequency of CD4^+^CD45RC^high^ was associated with cancer occurrence, we next analyzed if CD45RC expression on CD4^+^ and CD8^+^ T cells were associated. CD45RC expression on CD4^+^ and CD8^+^ T cells were positively and strongly correlated (p<0.0001, Fig 3). When analyzing outcomes in the cohort, we observed that cancer and AR occurrence tended to affect different patients (Fig 3), with only 2/89 patients experiencing both events (p = 0.085). Thus, these data suggest that AR and cancer risks are dissociated and may be assessed by determining CD45RC expression on T cells before transplantation.

## Discussion

In the present work, by studying a cohort of first time transplanted kidney recipients, we show that pre-transplant level of CD45RC expression on circulating T cells is associated with both cancer and AR occurrence. Indeed, after a mean follow-up of 11 years, the present work confirms our previously reported 5-years analysis, by showing that a high proportion of pre-transplant CD8^+^CD45RC^high^ T cells is associated with AR [23].

Moreover, we were able to confirm our hypothesis by showing in our population that a low proportion of CD4^+^CD45RC^high^ T cells was associated with cancer occurrence. In a predictive point of view and based on determined cut-offs, high frequency of CD8^+^CD45RC^high^ and low frequency of CD4^+^CD45RC^high^ T cells allowed to predict a >20-fold and a nearly 4-fold increased risk of developing AR and cancer, respectively, after adjustment on classical risk factors. Thus, this work supports that CD45RC expression on T cells may constitute a promising biomarker to assess both cancer and AR risks before transplantation. Interestingly, we observed a positive correlation between frequency of CD45RC^high^ on CD4^+^ and CD8^+^ T cells suggesting that, as a double-edged sword, patients prone to AR before transplantation also have a lower risk of experiencing cancer. CD45RC being a membrane-expressed molecule, its measurement on T cells by flow cytometry is simple and reproducible in our hands, fitting with the requirements for its use in routine.

Level of CD45RC expression on CD4^+^ and CD8^+^ T cells allows to differentiate T cell subsets with different in vitro profiles of cytokine production and proliferation ability. Indeed, in healthy humans, CD4^+^CD45RC^low^ T cells have a lower ability to proliferate as compared to CD4^+^CD45RC^high^ T cells [23]. While CD4^+^CD45RC^high^ subset produces both Th1 and Th2 type cytokines, CD45RC^low^ subset produces less amounts of Th1/Th2 cytokines and higher amounts of IL-17 [23, 24]. In respect to the CD8^+^ T cell compartment, CD45RC subsets have similar proliferative capacities, but CD45RC^high^ subset preferentially secretes Th1 cytokines including IFN-_**ϒ**_, while CD45RC^low^ subset produces mainly Th2 type cytokines [23, 24, 27].

We observed that patients with a low pre-transplant proportion of CD4^+^CD45RC^high^, or accordingly, patients with a high proportion of CD4^+^CD45RC^low^ T cells had a greater risk of developing cancer after transplantation. Given the in vitro cytokine profile of CD4^+^CD45RC^low^ T cells, this observation suggests that an unbalanced Th1/Th2 ratio in favor to a Th2 response may predispose to cancer development in kidney transplant recipients. Supporting this view, Th2-type cells infiltrating tumor and Th2 cytokines have been reported to favor tumor occurrence and development [16]. Given that regulatory T cells are contained within the CD45RC^low^ population, an additional interpretation of our results may be that patients with higher frequency of CD45RC^low^ T cells have greater frequency of regulatory T cells. Interestingly, Hoppe et al recently reported increased count and proportion of circulating regulatory T cells at cancer occurrence in kidney transplant patients [14]. In this work, the authors also observed that higher regulatory T cell count predicted the occurrence of invasive cancer in patients that previously experienced skin cancer [14].

Here, we also confirm our previous result that a high frequency of pre-transplant CD8^+^CD45RC^high^ T cells is strongly associated with AR occurrence. This suggests that CD45RC expression allows to identify a pool of naïve CD8^+^ T cells with greater allo-reactive properties. Supporting a critical role for CD45RC expression in allo-reactivity, Picarda et al recently showed that depletion of CD45RC^high^ T cells by using a specific monoclonal antibody allowed to induce tolerance in a rat cardiac allo-transplant model and in a humanized mice model of graft versus host disease [22]. In this model, CD45RC^low^ T cell subsets were not depleted, including regulatory T cells that are contained within the CD45RC^low^ subset [22, 23]. Thus, these results also suggest that the difference between CD45RC subsets to predict AR may be related to variations in regulatory T cell compartment between patients with high versus low CD45RC expression on T cells.

The present work highlights an issue that we consider of significant clinical interest. Indeed, we observed that patients of the cohort that developed AR tended to have a lower incidence of cancer. On the other hand, immunosuppressive treatments are associated with cancer development and lowering immunosuppressive treatments to improve long-term immunosuppression-related complications is part of the day to day practice [10]. However, such a strategy without a reliable stratification of risks may be deleterious and associated with increased AR occurrence [28]. We observed a very closed correlation between high expression of CD45RC on CD4^+^ and CD8^+^ T cells suggesting that patients at higher AR risk have a lower cancer risk. Based on our observations, patients with a low pretransplant frequency of CD45RC^high^ T cells which are at higher risk of cancer could benefit from immunosuppressive regimen alleviation, while patients with a high pretransplant frequency of CD45RC^high^ T cells which are at higher risk of AR could benefit from a stronger immunosuppressive regimen. Thus, determining CD45RC T cell phenotype before transplantation appears as a promising tool to achieve an accurate stratification of both AR and cancer risk in candidate patients to kidney transplantation.

Undeniably, our study has several limitations including the population size and its retrospective design. Interestingly, the population of the study fits well with patients we consider to be at low immunological risk nowadays. In the AR risk point of view, these patients are finally those that may benefit from an individualized immunosuppressive regimen. Of note, at the time of the study, induction therapy with anti-thymocyte antibodies and maintenance regimen with tacrolimus monotherapy was used in our center in an effort to minimize long-term immunosuppressive drug exposition [29].

In conclusion, pre-transplant CD45RC phenotyping of blood T cells appear as a promising biomarker to stratify the risk of cancer and AR in kidney transplant patients. Our results suggest that AR and cancer predisposition, at least within the first years following kidney transplantation, are linked to specific pre-transplant immune profiles. Thus, tailoring of the immunosuppressive regimens according to these immune profiles could allow to prevent post-transplant complications.

## Supporting information

S1 Fig Representative experiment of the flow cytometry gating strategy. (PDF)

S2 Fig Patient (A) and graft (B) survival of the cohort population.
(PDF)

S3 Fig Frequency of (A) CD4+CD45RChigh, (B-D) CD8+CD45RC subpopulations (high, int and low) according to the development (cancer, AR or both) or not of posttransplant outcomes. Results are expressed as medians and 95CI intervals. Statistical analyses were done using Kruskal-Wallis test with multiple comparisons.
(PDF)

S4 Fig ROC curve analysis of CD4^+^CD45RC^high^ (red line) and CD8^+^CD45RC^high^ (blue line) T cells for posttransplant death. (PDF)

S1 Table Baseline characteristics of the population. (DOCX)

S2 Table Frequency of CD4^+^ and CD8^+^CD45RC subsets according to cancer subtype. Results are expressed as the % of subset among CD4^+^ or CD8^+^ T cells.
(DOCX)

S3 Table Univariate analysis of factors associated with acute rejection occurrence. (DOCX)

S4 Table Multivariate cox analysis for acute rejection prediction. (DOCX)

S5 Table Multivariate cox analysis for posttransplant death. (DOCX)

## Abbreviations

AMR: antibody-mediated rejection
AR: acute rejection
DSA: Donor specific anti-HLA antibodies
PTLD: post-transplant lymphoproliferative disease
ROC: receiver operating characteristics
TCMR: T cell-mediated rejection
Treg: regulatory T cells
