# Supplementary figures and images for: Pre-transplant CD45RC expression on blood T cells differentiates patients with cancer and rejection after kidney transplantation
Source: PLoS One. 2019 Mar 29;14(3):e0214321. doi: 10.1371/journal.pone.0214321 (PMC6440623; doi:10.1371/journal.pone.0214321)

Figure S1

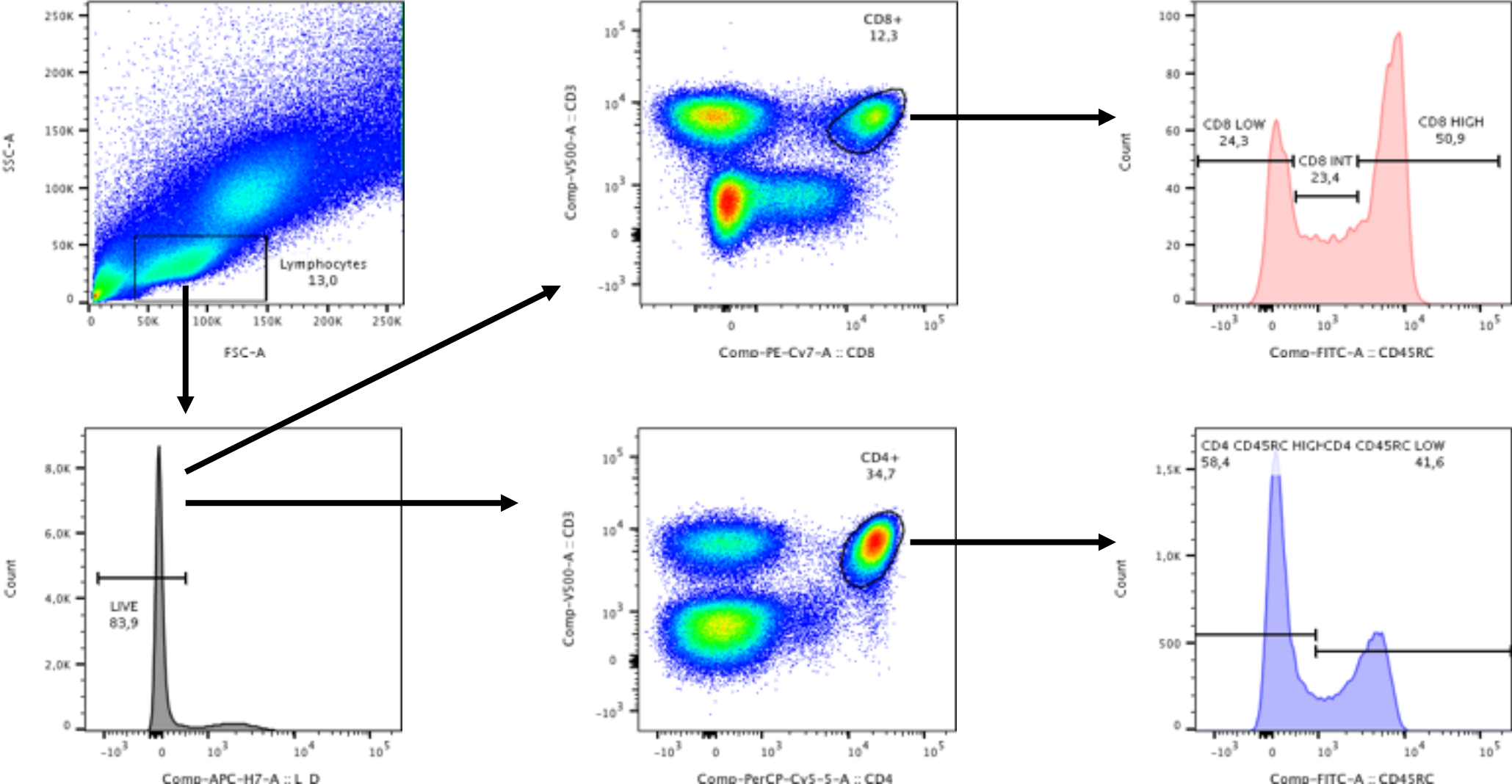

Supplement: S1 Fig — (PDF) [file pone.0214321.s001.pdf]

**A**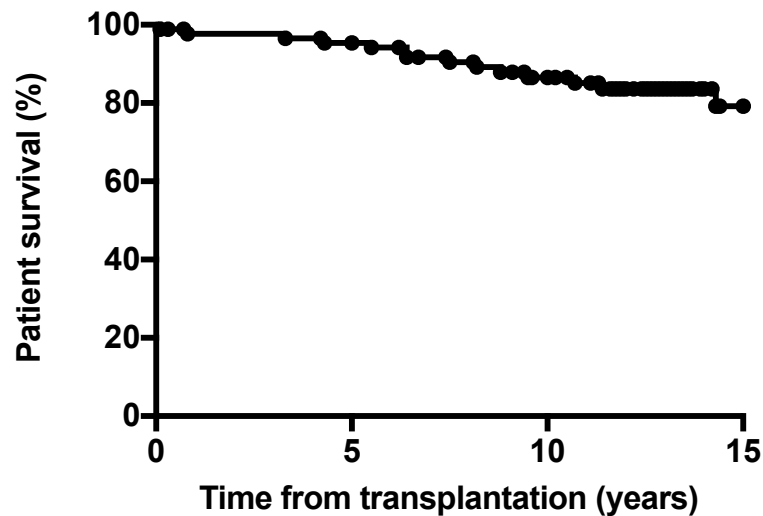

At risk 89 (100) 80 (95.4) 64 (86.6) 14 (79.2)

**B**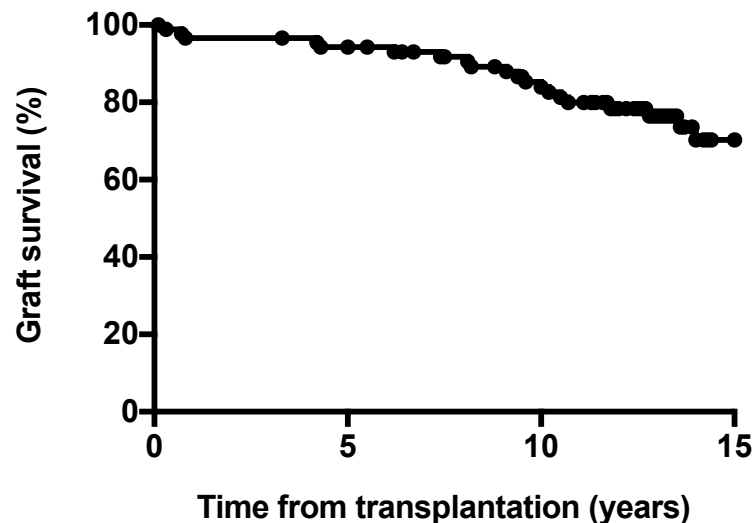

At risk 89 (100) 80 (94.3) 64 (84) 14 (70.3)

Supplement: S2 Fig — Patient (A) and graft (B) survival of the cohort population. (PDF) [file pone.0214321.s002.pdf]

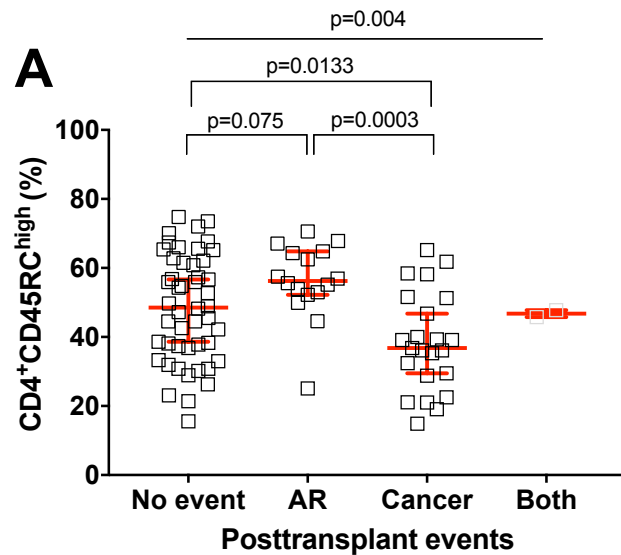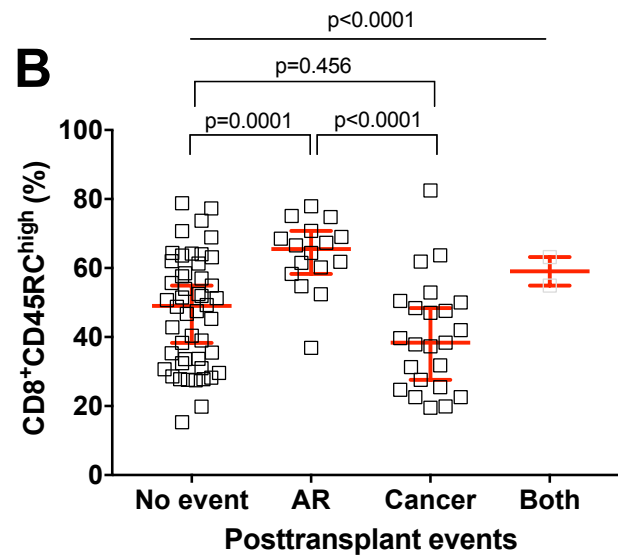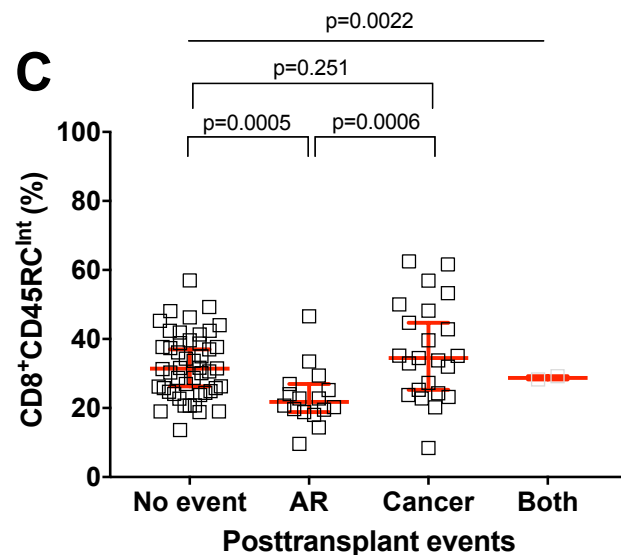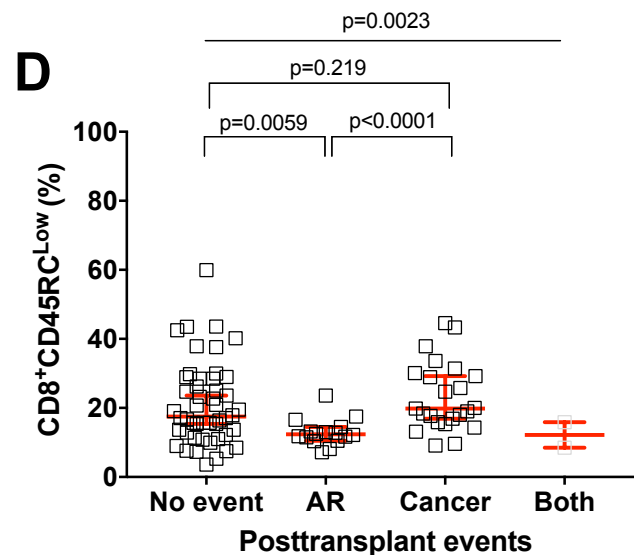

Supplement: S3 Fig — Frequency of (A) CD4+CD45RChigh, (B-D) CD8+CD45RC subpopulations (high, int and low) according to the development (cancer, AR or both) or not of posttransplant outcomes. Results are expressed as medians and 95CI intervals. Statistical analyses were done using Kruskal-Wallis test with multiple comparisons. (PDF) [file pone.0214321.s003.pdf]

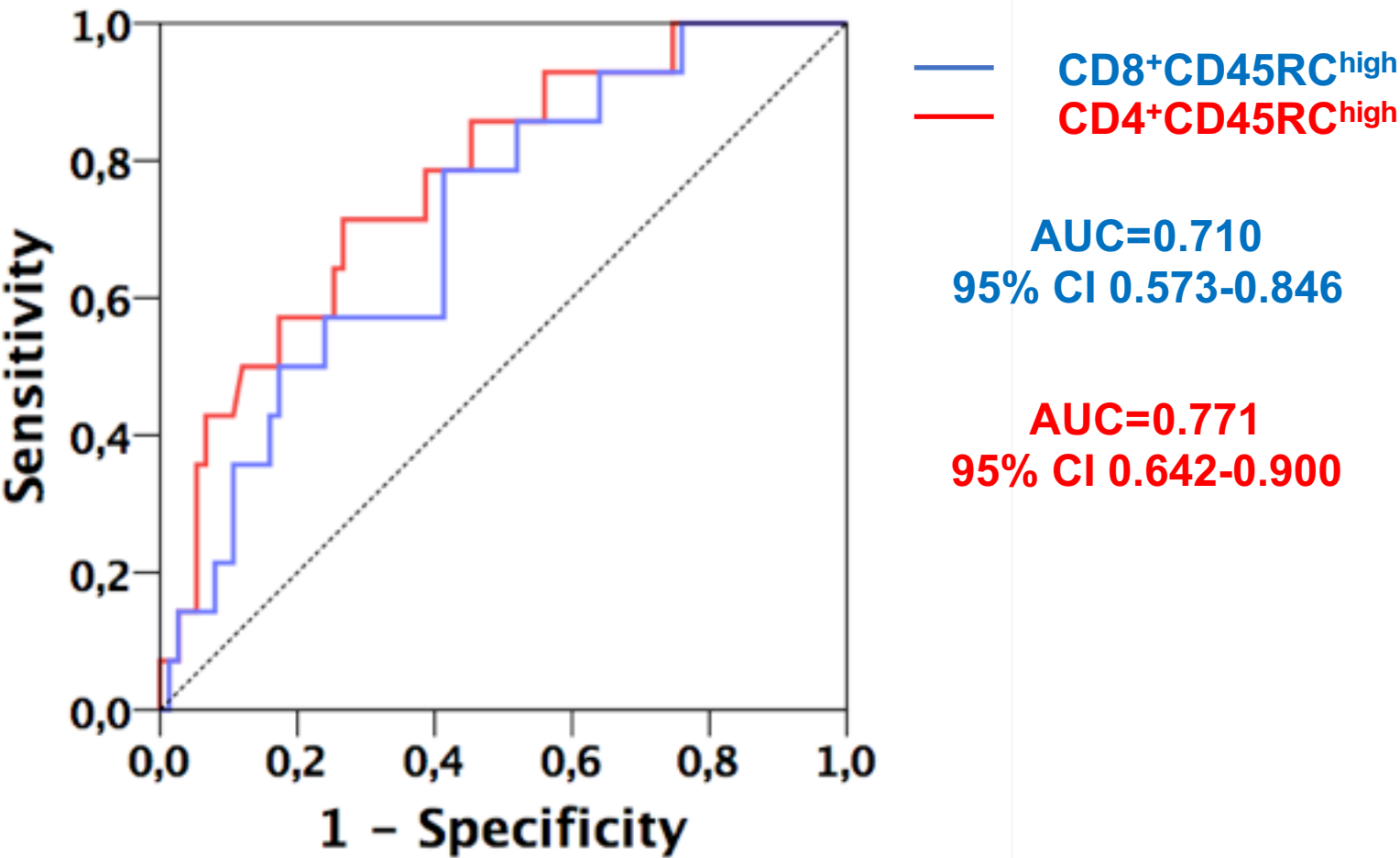

Supplement: S4 Fig — (PDF) [file pone.0214321.s004.pdf]
