# Supplementary material for: Pre-transplant CD45RC expression on blood T cells differentiates patients with cancer and rejection after kidney transplantation
Source: PLoS One. 2019 Mar 29;14(3):e0214321. doi: 10.1371/journal.pone.0214321 (PMC6440623; doi:10.1371/journal.pone.0214321)
Supplement: S1 Table — (DOCX) [file pone.0214321.s005.docx]

**Table S1. Baseline characteristics of the population**

|  | **All patients** |  |
| --- | --- | --- |
|  | **(n=89)** |  |
| **Baseline characteristics** |  |  |
| Sex (M/F) | 69/20 |  |
| Age (years) | 48.1 ± 15.2 |  |
| Weigh (kg) | 70.0 ± 16.1 |  |
| BMI (Kg/m^2^) | 23.8 ± 5.5 |  |
| Original nephropathy, n (%) |  |  |
| ADPKD | 17 (19.1) |  |
| IgA nephropathy | 20 (22.5) |  |
| Other GN | 20 (22.5) |  |
| TIN/urologic | 18 (20.2) |  |
| Vascular nephropathy | 10 (11.2) |  |
| Undetermined nephropathy | 4 (4.5) |  |
| **History of transplantation** |  |  |
| Pre-transplant dialysis, n (%) | 69 (77.5) |  |
| Donor age, years | 41.0 ± 17.2 |  |
| Cold ischemia time (hours) | 18.3 ± 5.9 |  |
| HLA mismatch, n |  |  |
| HLA A&B | 2.5 ± 0.7 |  |
| HLA DR | 1.2 ± 0.6 |  |
| **Immunosuppressive regimens** |  |  |
| Induction therapy |  |  |
| None, n (%) | 8 (9.0) |  |
| Basiliximab, n (%) | 26 (29.2) |  |
| Antithymocyte globulins, n (%) | 55 (61.8) |  |
| Maintenance regimen |  |  |
| Tac-based, n (%) | 76 (85.4) |  |
| Cyclosporin-based, n (%) | 13 (14.6) |  |
| Tac monotherapy at month 6, n (%) | 57 (64.0) |  |

ADPKD, autosomic dominant polycystic kidney disease ; BMI, body mass index ; GN, glomerulonephritis ; HLA, human leukocyte antigens ; Tac, Tacrolimus ; TIN, Tubulo-interstitial nephropathy.
