# Supplementary material for: Pre-transplant CD45RC expression on blood T cells differentiates patients with cancer and rejection after kidney transplantation
Source: PLoS One. 2019 Mar 29;14(3):e0214321. doi: 10.1371/journal.pone.0214321 (PMC6440623; doi:10.1371/journal.pone.0214321)
Supplement: S2 Table — Results are expressed as the % of subset among CD4+ or CD8+ T cells. (DOCX) [file pone.0214321.s006.docx]

**Table S2. Frequency of CD4^+^ and CD8^+^CD45RC subsets according to cancer subtype.** Results are expressed as the % of subset among CD4^+^ or CD8^+^ T cells.

|  | **Yes** | **No** | ***p*** |  |
| --- | --- | --- | --- | --- |
|  |  |  |  |  |
| **Non melanoma skin cancers, n** | 17 | 72 |  |  |
| CD4^+^ CD45RC ^high^ | 38.4 ± 13.4 | 49.3 ± 15.1 | **0.007** |  |
| CD8^+^ CD45RC ^high^ | 38.7 ± 12.7 | 51.1 ± 16.8 | **0.006** |  |
| CD8^+^ CD45RC ^int^ | 36.2 ± 13.3 | 30.5 ± 10.7 | 0.064 |  |
| CD8^+^ CD45RC ^low^ | 25.1 ± 10.5 | 18.8 ± 10.7 | **0.031** |  |
| **Solid and PTLD, n** | 12 | 77 |  |  |
| CD4^+^ CD45RC ^high^ | 37.3 ± 14.5 | 48.8 ± 14.9 | **0.015** |  |
| CD8^+^ CD45RC ^high^ | 47.4 ± 18.9 | 48.9 ± 16.5 | 0.779 |  |
| CD8^+^ CD45RC ^int^ | 35.2 ± 15.3 | 31.1 ± 10.7 | 0.239 |  |
| CD8^+^ CD45RC ^low^ | 17.3 ± 7.4 | 20.4 ± 11.3 | 0.363 |  |
