# Supplementary material for: Pre-transplant CD45RC expression on blood T cells differentiates patients with cancer and rejection after kidney transplantation
Source: PLoS One. 2019 Mar 29;14(3):e0214321. doi: 10.1371/journal.pone.0214321 (PMC6440623; doi:10.1371/journal.pone.0214321)
Supplement: S3 Table — (DOCX) [file pone.0214321.s007.docx]

**Table S3. Univariate analysis of factors associated with acute rejection occurrence.**

|  | **Acute rejection**  **(n=18)** | **No rejection**  **(n=71)** | ***p*** |  |
| --- | --- | --- | --- | --- |
|  |  |  |  |  |
| **Baseline characteristics** |  |  |  |  |
| Sex (M/F) | 14/4 | 55/16 | 1.000 |  |
| Age (years) | 37.2 ± 16.3 | 50.9 ± 13.7 | $\boldsymbol{<}$**0.001** |  |
| **History of transplantation** |  |  |  |  |
| Pre-transplant dialysis, n (%) | 14 (77.7) | 55 (74.5) | 0.977 |  |
| Donor age, years | 34.4 ± 18.0 | 42.7 ± 16.7 | 0.068 |  |
| Cold ischemia time (hours) | 17.2 ± 7.4 | 18.6 ± 5.5 | 0.393 |  |
| HLA mismatch (ABDR) | 3.78 ± 1.4 | 3.85 ± 1.2 | 0.837 |  |
| **Immunosuppressive regimens** |  |  |  |  |
| Induction therapy (none/basiliximab/ATG) | 0/8/10 | 8/18/45 | 0.138 |  |
| Tac monotherapy, n | 11 | 46 | 0.772 |  |
| Tacrolimus-based regimen, n | 16 | 60 | 1.000 |  |
