# Supplementary material for: Pre-transplant CD45RC expression on blood T cells differentiates patients with cancer and rejection after kidney transplantation
Source: PLoS One. 2019 Mar 29;14(3):e0214321. doi: 10.1371/journal.pone.0214321 (PMC6440623; doi:10.1371/journal.pone.0214321)
Supplement: S4 Table — (DOCX) [file pone.0214321.s008.docx]

**Table S4. Multivariate cox analysis for acute rejection prediction.**

|  | **Multivariate Cox models** | **HR** | **95% CI** | ***P*** |
| --- | --- | --- | --- | --- |
| **CD4^+^CD45RC^high^** | CD4 CD45RC^high^ (>44.5%) | 9.88 | 1.21-80.5 | **0.032** |
|  | Age at transplantation* | 0.97 | 0.94-1.00 | 0.136 |
|  | Gender (male) | 1.28 | 0.83-3.85 | 0.659 |
|  | Induction (ATG) | 0.76 | 0.36-3.40 | 0.592 |
|  |  |  |  |  |
|  |  |  |  |  |
| **CD8^+^CD45RC^high^** | CD8 CD45RC^high^ (>52.1%) | 21.7 | 2.67-176.2 | **0.004** |
|  | Age at transplantation* | 0.98 | 0.95-1.01 | 0.332 |
|  | Gender (male) | 1.13 | 0.37-3.49 | 0.827 |
|  | Induction (ATG) | 0.54 | 0.20-1.46 | 0.224 |
|  |  |  |  |  |

* per year increment
