# Supplementary material for: Pre-transplant CD45RC expression on blood T cells differentiates patients with cancer and rejection after kidney transplantation
Source: PLoS One. 2019 Mar 29;14(3):e0214321. doi: 10.1371/journal.pone.0214321 (PMC6440623; doi:10.1371/journal.pone.0214321)
Supplement: S5 Table — (DOCX) [file pone.0214321.s009.docx]

**Table S5. Multivariate cox analysis for death prediction.**

|  | **Multivariate Cox models** | **HR** | **95% CI** | ***P*** |
| --- | --- | --- | --- | --- |
| **CD4^+^CD45RC^high^** | CD4 CD45RC^high^ (<38.8%) | 2.45 | 0.742-8.09 | 0.141 |
|  | Age at transplantation* | 1.14 | 1.07-1.22 | **<0.001** |
|  | Gender (male) | 1.02 | 0.26-4.02 | 0.981 |
|  | Induction (ATG) | 2.33 | 0.58-9.35 | 0.230 |
|  |  |  |  |  |
|  |  |  |  |  |
| **CD8^+^CD45RC^high^** | CD8 CD45RC^high^ (<49.1%) | 1.89 | 0.47-7.64 | 0.562 |
|  | Age at transplantation* | 1.14 | 1.07-1.21 | **<0.001** |
|  | Gender (male) | 1.52 | 0.37-6.37 | 0.562 |
|  | Induction (ATG) | 2.23 | 0.56-8.85 | 0.255 |
|  |  |  |  |  |

* per year increment
